# Cytotoxic Effects of Dimorfolido-*N*-Trichloroacetylphosphorylamide and Dimorfolido-*N*-Benzoylphosphorylamide in Combination with C_60_ Fullerene on Leukemic Cells and Docking Study of Their Interaction with DNA

**DOI:** 10.1186/s11671-017-1893-3

**Published:** 2017-02-17

**Authors:** S. Prylutska, I. Grynyuk, A. Grebinyk, V. Hurmach, Iu. Shatrava, T. Sliva, V. Amirkhanov, Yu. Prylutskyy, O. Matyshevska, M. Slobodyanik, M. Frohme, U. Ritter

**Affiliations:** 1 Taras Shevchenko National University of Kyiv, 64 Volodymyrska Str., Kyiv, 01601 Ukraine; 2 Technical University of Applied Sciences of Wildau, 1 Hochschulring Str., Wildau, 15745 Germany; 3 Technical University of Ilmenau, 25 Weimarer Str., Ilmenau, 98693 Germany

**Keywords:** Dimorfolido-*N*-trichloroacetylphosphorylamide, Dimorfolido-*N*-benzoylphosphorylamide, C_60_ fullerene, Leukemic cells, DNA, Computer modeling

## Abstract

Dimorfolido-*N*-trichloroacetylphosphorylamide (HL1) and dimorfolido-*N*-benzoylphosphorylamide (HL2) as representatives of carbacylamidophosphates were synthesized and identified by the methods of IR, ^1^H, and ^31^P NMR spectroscopy. In vitro HL1 and HL2 at 1 mM concentration caused cell specific and time-dependent decrease of leukemic cell viability. Compounds caused the similar gradual decrease of Jurkat cells viability at 72 h (by 35%). HL1 had earlier and more profound toxic effect as compared to HL2 regardless on leukemic cell line. Viability of Molt-16 and CCRF-CEM cells under the action of HL1 was decreased at 24 h (by 32 and 45%, respectively) with no substantial further reducing up to 72 h. Toxic effect of HL2 was detected only at 72 h of incubation of Jurkat and Molt-16 cells (cell viability was decreased by 40 and 45%, respectively).

It was shown that C_60_ fullerene enhanced the toxic effect of HL2 on leukemic cells. Viability of Jurkat and CCRF-CEM cells at combined action of C_60_ fullerene and HL2 was decreased at 72 h (by 20 and 24%, respectively) in comparison with the effect of HL2 taken separately.

In silico study showed that HL1 and HL2 can interact with DNA and form complexes with DNA both separately and in combination with C_60_ fullerene. More stable complexes are formed when DNA interacts with HL1 or C_60_ + HL2 structure. Strong stacking interactions can be formed between HL2 and C_60_ fullerene. Differences in the types of identified bonds and ways of binding can determine distinction in cytotoxic effects of studied compounds.

## Background

Nowadays, it is important to create new biocompatible nanomaterials that exhibit pharmacological properties, antitumor activity, and modulate the biological effects of chemotherapeutic drugs.

Carbacylamidophosphates as the structural analog of β-diketones, that are promising class of biologically active compounds with antimitotic and antiproliferative activities, can be used as antitumor agents [1–3].

In previous study with the use of in silico analysis, we have shown that dimethyl-*N*-(benzoyl)amidophosphate as representative of carbacylamidophosphates possesses an antitumor activity and interacts with DNA [4, 5].

In vitro toxic effects (decrease of cell viability and increase of ROS production) of 2.5 mM dimethyl-*N*-(benzoyl)amidophosphate on leukemic L1210 cells were demonstrated. It was shown that toxic effects of dimethyl-*N*-(benzoyl)amidophosphate on leukemic cells could be facilitated by C_60_ fullerene [5].

C_60_ fullerene is a chemically stable carbon nanostructure, able to interact with biomolecules and penetrate through plasma membrane inside the cell [6–8] and, thus, it can be used in biomedical applications [9–13]. C_60_ fullerene can form complexes with chemotherapeutic drugs such as doxorubicin, cisplatin, and paclitaxel, reducing their cytotoxic effect and enhancing the therapeutic effect [14–18].

**Fig. 1:**
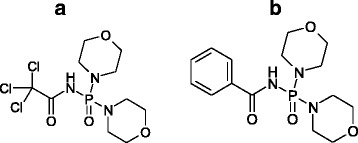
Carbacylamidophosphates. **a** Dimorfolido-*N*-trichloroacetylphosphorylamide (HL1). **b** Dimorfolido-*N*-benzoylphosphorylamide (HL2)

Two carbacylamidophosphate derivatives with different substituents were used in this study: dimorfolido-*N*-trichloroacetylphosphorylamide (HL1) contained CCl_3_ group (Fig. 1a) and dimorfolido-*N*-benzoylphosphorylamide (HL2)—benzene ring (Fig. 1b) at carbamide group.

The aim was to study the leukemic cell viability under the action of HL1 and HL2 separately and in combination with C_60_ fullerene and to estimate their interaction with DNA in silico.

## Methods

### Chemicals

MTT [3-(4,5-dimethylthiazol-2-yl)-2,5-diphenyl tetrazolium bromide], DMSO (Sigma-Aldrich Co, Ltd, USA).

### Characterization of Chemical Compounds

HL1 and HL2 were synthesized at the Department of Inorganic Chemistry, Chemical Faculty of Taras Shevchenko National University of Kyiv according to methods described earlier [19]. The composition of the synthesized compounds was confirmed by NMR and IR spectroscopy and measuring the melting points. The purity of above compounds was ≥98%.

IR measurements were performed on a *UR-10* and a Perkin–Elmer Spectrum BX spectrometer on samples as KBr pellets. ^1^H and ^31^P NMR spectra in DMSO-d_6_ and methanol-d_4_ solutions were obtained on a *Varian 400* NMR spectrometer at room temperature. Chemical shifts are referenced to SiMe_4_ as interior standard for ^1^H NMR and H_3_PO_4_ as exterior standard for ^31^P NMR.

HL1: m. p. 205 °C; IR (KBr, cm^–1^): *ν* = 3013 (NH), 1728 (CO), 1201 (PO); ^1^H NMR (DMSO-d_6_): 3.61 (m, 8H, CH_2λ_), 3.17 (m, 8H, CH_2β_).

HL2: m. p. 178 °C; (KBr, cm^–1^): *ν* = 3100 (NH), 1685 (CO), 1200 (PO); ^1^H NMR (methanol-d_4_): 3.36 (m, 8H, CH_2λ_), 3.24 (m, 8H, CH_2β_), 7.49, 7.56, 7.90 (m, C_6_H_5_) (8:8:5:1).

The synthesized compounds were dissolved in 10% DMSO to a final concentration of 0.05 M.

### Characterization of C_60_ Fullerene Aqueous Solution

A highly stable aqueous colloid solution of C_60_ fullerene (10^−4^ M, purity >99.5%, nanoparticle average size up to 50 nm) was synthesized in Technical University of Ilmenau (Germany) as described in [20, 21].

### In Vitro Study

#### Cell Culture

The experiments were done on different human acute T cell leukemic cell lines. Cell lines were purchased from the Leibniz Institute DSMZ-German Collection of Microorganisms and Cell Cultures: Jurkat (ACC 282), CCRF-CM (ACC 240), and Molt-16 (ACC 29). The cells were cultured in RPMI 1640 medium supplemented with 10% FBS, 1% penicillin/streptomycin, and 2 mM gluthamine using 25-cm^2^ flasks at 37 °C with 5% CO_2_ in humidified incubator.

Cells in RPMI 1640 medium were preincubated with C_60_ fullerene (10^−5^ M) during 1 h. After that, HL1 or HL2 was added to above medium and incubated for 24, 48, and 72 h.

Cell survival without addition of chemical compounds and C_60_ fullerene was received as 100% (control sample contained 0.05 M DMSO).

### Cell Viability (MTT) Assay

Cell viability was assessed by the MTT reduction assay [22]. At indicated time points of incubation, 200 μl aliquots (1 × 10^5^ cells) was placed into the 96-well microplates, 20 μl of MTT solution (4 mg/ml) was added to each well, and the plates were incubated for another 2 h. The culture medium was then replaced with 100 μl of DMSO; diformazan formation was determined by measuring absorption at 570 nm with a microplate reader Tecan Infinite M200 Pro (Switzerland).

### In Silico Study

The double-helix DNA molecule was used as a template from PDB (Protein Data Bank) base. The interactions of DNA molecule with HL1 or HL2 separately and in combination with C_60_ fullerene have been studied. We took into consideration the following structures of DNA molecule: 2MIW (CCATCGCTACC—intercalation of compound into a small groove of DNA helix), 1XRW (CCTCGTCC—intercalation of compound into a small groove of DNA helix), and 2M2C (GCGCATGCTACGCG—binding of compound with large and small grooves of DNA helix). We applied the algorithm of systematical docking (SDOCK+), built-in the QXP package (this method demonstrates all possible conformations of the studied structures with the minimal value of root mean square deviation (Rmsd)) [23]. To each compound (HL1 or HL2) in combination with the C_60_ fullerene, we generated 300 potentially possible complexes with DNA, the 10 best of which were selected for the next stage, using a scoring function, built-in the QXP package [24].

The interactions of the DNA molecule with HL1 or HL2 separately and in combination with the C_60_ fullerene were characterized by the following parameters: (1) the number of hydrogen bonds, (2) the area of contacting surfaces of DNA and corresponding structure, (3) the distance between the DNA and docked structure, and (4) the total energy of the binding structure.

To assess the stability of the complexes of chemical compounds with C_60_ fullerene, we conducted the short-molecular dynamics (MD, 25 ps) using a Nosé-Poincaré-Anderson algorithm (NPA) [25, 26]. The calculations were performed on the following parameters: temperature (in K)—300; pressure (in kPa)—100; binding involving the hydrogen atom or ligand was limited by the algorithm [26].

### Statistical Analysis

The data were represented as M ± SD of more than five independent experiments. Mean (M) and standard deviation (SD) were calculated for each group. Statistical analysis was performed using two-way ANOVA followed by post Bonferroni’s tests. A value of *p* < 0.05 was considered statistically significant. Data processing and plotting were performed by IBM PC using specialized applications GraphPad Prism 7 (GraphPad Software Inc., USA).

## Results and Discussion

In the structures of HL1 and HL2, the carbonyl and phosphoryl groups are in *anti*-positions to each other as in most carbacylamidophosphates. In the structure of HL1, the bond lengths C–O (1.202(4) A) and C–N (1.346(4) A) are influenced by the substituent nature near the carbonyl group and have typical values for trichloracetylamide derivatives. The carbon atom of CCl_3_ group has a tetrahedral environment (Fig. 1a). For HL2, the planar benzene ring is rotated relative to the plane of the carbamide group on angle 22.34(27)°, which does not exclude the possibility of π-interaction between the benzene ring and the (O)CNP(O) fragment (Fig. 1b). It should be noted a close contact between the electrophilic phosphorus and nucleophilic oxygen atoms of the carbonylic group for both ligands. The distance between abovementioned atoms 3.02 A for HL1 and 3.122(3) A for HL2 is slightly below than the sum of the Van der Waals radii of phosphorus and oxygen atoms (3.3 A) that can be considered as an evidence of estimate covalent contribution in interatomic interaction [27, 28].

More electronegative character of CCl_3_ group in comparison with C_6_H_5_ (phenyl) group reflects in the shortening of intramolecular hydrogen bonds N···O and O…H type: for HL1—1.81(1) A and 2.73(1) A, and for HL2—2.103(3) A and 2.946(3) A, respectively.

HL1 and HL2 at 1 mM concentration were screened for their toxicity against human T cell leukemic cells Jurkat, CCRF-CEM, and Molt-16 using MTT assay.

**Fig. 2:**
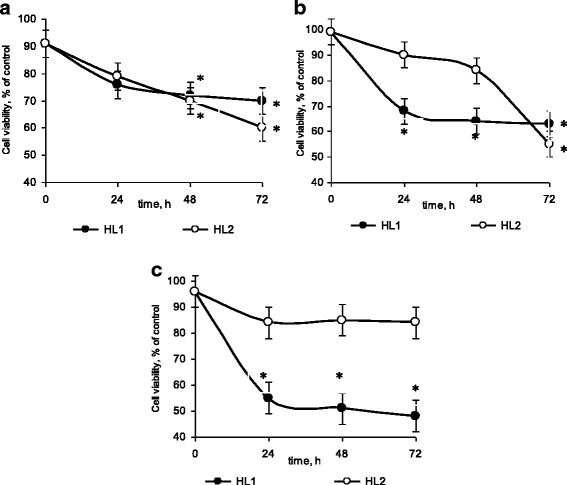
Viability of leukemic cells in the presence of 1 mM HL1 or HL2. M ± m, *n* = 8; *p < 0.05 compared to control cells. **a** Jurkat cells. **b** Molt-16 cells. **c** CCRF-CEM cells

It was shown that HL1 and HL2 decrease the viability of leukemic cells. The observed toxic effect was cell specific and time dependent (Fig. 2).

HL1 and HL2 caused the similar gradual decrease of Jurkat cells viability by about 35% at 72 h (Fig. 2a).

The dynamics of Molt-16 and CCRF-CEM cell death under the action of HL1 was similar: cell viability was decreased at 24 h with no substantial further reducing up to 72 h. But the value of toxic effect was higher on CCRF-CEM cells (52% at 72 h) as compared with Molt-16 cells (37% at 72 h) (Fig. 2b, c).

Toxic effect of HL2 against Molt-16 cells was less pronounced as compared with HL1. Molt-16 cells viability was decreased (by 45%) only in 72 h, while no substantial decrease of CCRF-CEM cell viability was detected (Fig. 2b, c).

The obtained data enabled us to conclude that HL1 had earlier and more profound toxic effect as compared to HL2 regardless of leukemic cell lines. Toxic effect of HL2 was detected only at 72 h of incubation of Jurkat and Molt-16 cells (Fig. 2a, b).

The higher HL1 toxic effect could be connected with the presence in its structure of Cl atoms, which possess alkylating potential and are the constituents of chemotherapeutic drug such as cisplatin [29].

The high tumor-specific toxicity of β-diketones derivatives was confirmed by evaluating their effects against human carcinoma cell lines HSC-3 (oral squamous), HSG (submandibular gland), and HL-60 (promyelocytic leukemia) as well [1].

It was shown that β-diketones exist mainly in the enolic form and form metal chelates with Fe, Cu, and Zn ions. Recent studies suggest that metal chelates induce apoptotic cell death in various tumor cell lines and they are potential antitumor agents against malignant melanomas [30, 31].

To estimate the possibility to enhance the toxicity of studied compounds, their combined action with C_60_ fullerene on leukemic cells was investigated.

It was shown that C_60_ fullerene (10^-5^ M) does not affect the viability of leukemic cells during incubation period (data not shown).

No increase of HL1 toxicity was detected when Jurkat, Molt-16, or CCRF-CEM cells were preincubated with C_60_ fullerene (data not shown).

**Fig. 3:**
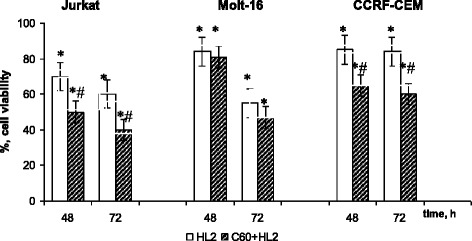
Viability of leukemic cells at the combined action of 1 мM HL2 and C_60_ fullerene. M ± m, *n* = 8; *p < 0.05 compared to control cells; #*p* < 0.05 compared to HL2

At combined action of C_60_ fullerene and HL2, an enhanced toxic effect in comparison with the effect of HL2 taken separately was observed. In this case, a viability of Jurkat and CCRF-CEM cells was additionally decreased by 20 and 24% at 72 h, respectively (Fig. 3).

At the same time, no enhancement of HL2 toxic effect under the combined action with C_60_ fullerene against Molt-16 cells was found (Fig. 3).

In vitro experiments combined with modeling simulation have shown that C_60_ fullerene is significantly accumulated in human leukemic K562 cells since it could not be efficiently effluxed by P-gp protein [32]. So, C_60_-induced enhancement of HL2 cytotoxicity could be triggered by C_60_ fullerene intracellular interactions at the level of membrane phospholipids, cytosolic proteins, DNA, and other biological structures. It is well known that nanoparticles can interact with organic molecules using van der Waals forces, hydrophobicity, π-interactions, and enthalpy driven [33]. Previously with the use of molecular modeling, we have shown that stable C_60_-DNA complex is formed after C_60_ fullerene binding with DNA [5].

Computer simulation of HL1 and HL2 interactions with DNA was used to estimate the possible nature of their bonds with biological molecules and the ability of C_60_ fullerene to modify such interactions.

**Fig. 4:**
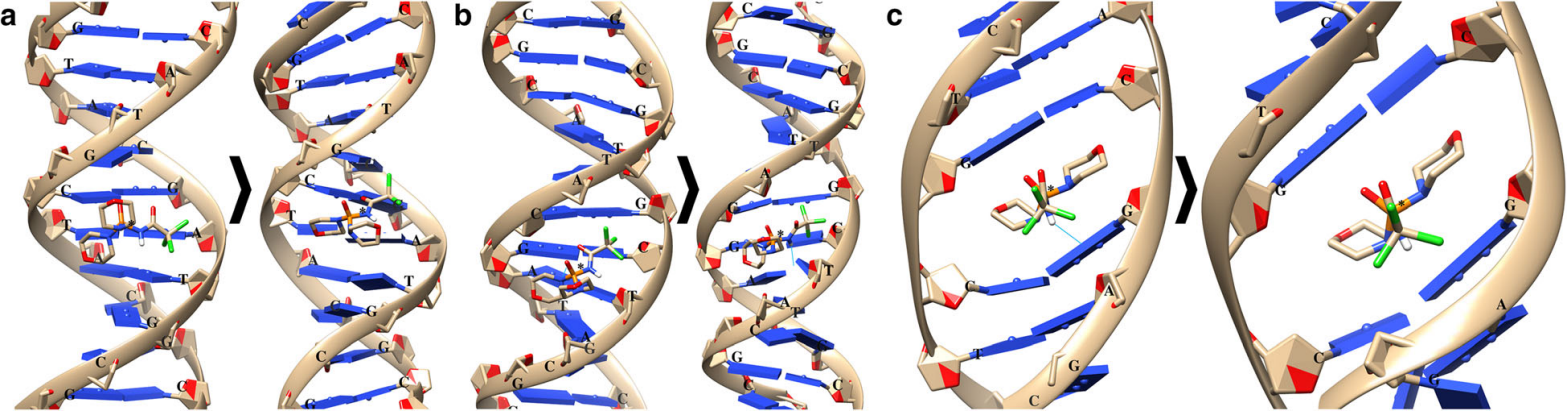
The interaction of DNA molecule with HL1: **a** and **b**—binding with small and large grooves; **c**—intercalation into a small groove. The used DNA structures of the PDB database: **a** and **b**—2M2C; **c**—1XRW

It was shown that HL1 forms the stable complexes with DNA when bound with a large groove and during the intercalation into a small groove. So, when the binding shift of DNA double helix was 3.07 Å, and for HL1, it equals to 1.8 Å; at intercalation, the DNA molecule shifted to 3.24 Å, and HL1—2.24 Å (Fig. 4).

According to energy parameters, the formed complexes are not rigid (Table. 1). So, when binding HL1 with a large groove, the contact energy is −34.8 kJ/mol, and at intercalation into DNA, it is −61.0 kJ/mol; the steric clashes between DNA and HL1 in these cases are 1.4 and 7.7 kJ/mol, respectively.

**Table 1:** The energy parameters (in kJ/mol) of interaction of the studied structures with DNA double helix

Structure	The energy parameters				
	FreE	Cntc	Hbnd	Bump	Int
The binding with a large groove					
HL1	−1.8	−34.8	−0.4	1.4	4.0
HL2	−3.6	−41.7	−1.8	1.6	3.7
HL1 + C_60_	−20.3	−70.5	−1.1	7.8	3.1
HL2 + C_60_	−20.3	−66.8	0.0	6.5	3.9
The binding with a small groove					
HL1	−4.9	−49.0	−2.7	3.9	4.8
HL2	−4.7	−61.1	0.0	11.2	7.2
HL1 + C_60_	−24.0	−87.8	0.0	11.2	5.3
HL2 + C_60_	−31.1	−84.3	−0.8	6.3	3.8
The intercalation into a small groove					
HL1	−7.0	−61.0	−1.7	7.7	6.8
HL2	2.3	−63.0	0.0	12.6	10.4
HL1 + C_60_	−22.5	−107.0	−1.4	19.6	7.2
C_60_ + HL1	−22.8	−97.1	0.0	17.1	4.2
HL2 + C_60_	−21.8	−100.0	0.0	18.6	5.1
C_60_ + HL2	−26.5	−89.9	−2.4	12.7	4.2

*FreE* the total energy of binding DNA and related structure, *Cntc* the contact energy of interacting compounds (the related structure with DNA), *Hbnd* the energy of hydrogen interactions, *Bump* the energy of steric clashes between DNA and build-in structure, *Int* the energy of steric clashes between the atoms of build-in structure

**Fig. 5:**
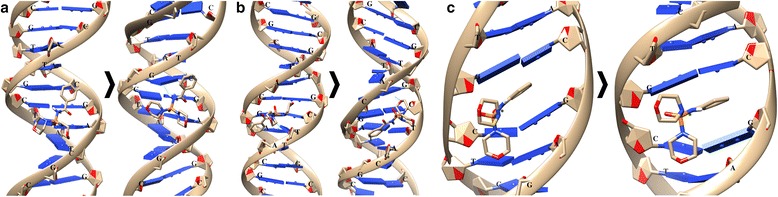
The interaction of DNA molecule with HL2: **a** and **b**—binding with small and large grooves; **c**—intercalation into a small groove. The used DNA structures of the PDB database: **a** and **b**—2M2C; **c**—1XRW

It is shown that HL2 forms the stable complexes with DNA only when bound with a small groove (Fig. 5a). The shift of DNA is 2.09 Å, and for HL2—1.45 Å.

When binding HL1 as well as HL2, observe no changes in their nucleotide environment (only insignificant movements in space take place).

**Fig. 6:**
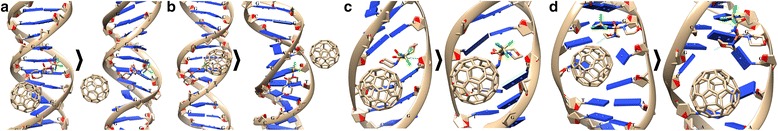
The interaction of DNA molecule with HL1in combination with the C_60_ fullerene (HL1 + C_60_ or C_60_ + HL1): **a** and **b**—binding with small and large grooves and **c** and **d**—intercalation into a small groove. The used DNA structures of the PDB database: **a** and **b**—2M2C; **c**—1XRW; and **d**—2MIW

**Fig. 7:**
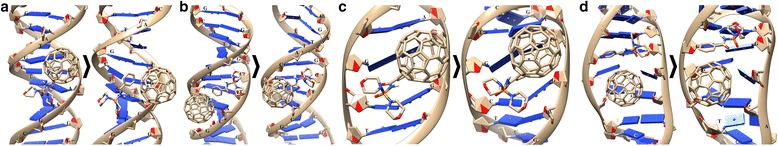
The interaction of DNA molecule with HL2 in combination with C_60_ fullerene (HL2 + C_60_ or C_60_ + HL2): **a** and **b**—binding with small and large grooves and **c** and **d**—intercalation into a small groove. The used DNA structures of the PDB database: **a** and **b**—2M2C; **c**—1XRW; and **d**—2MIW

The interaction of the DNA molecule with HL1 or HL2 in combination with C_60_ fullerene was studied depending on the sequence of interactions. If initially C_60_ fullerene binds with DNA, and then HL1 or HL2, the formed structure is designated as C_60_ + HL1 or C_60_ + HL2; otherwise—HL1 + C_60_ or HL2 + C_60_. We considered two options of the binding-studied structures with DNA molecule, namely with small and large grooves, and also the possible intercalation of these structures into the small groove (Figs. 6 and 7). The calculated energy parameters of the interaction of these structures with DNA molecule are given in Table 1.

HL1 + C_60_ or C_60_ + HL1: at binding HL1 + C_60_ structure with small and large grooves of DNA, there is a significant shift of the C_60_ fullerene compared to HL1 compound: Rmsd value is 5.22 Å in a small groove, and in a large groove—8.08 Å; meanwhile, Rmsd value for HL1 is 2.92 and 2.93 Å in small and large grooves, respectively (Fig. 6a, b).

At intercalation of the studied structures into a small groove of the DNA molecule, the significant values of Rmsd for C_60_ fullerene in the cases of HL1 + C_60_ (5.72 Å) and C_60_ + HL1 (6.42 Å) are also observed. Due to such shift of C_60_ molecule, a partial displacement of HL1 compound (Rmsd value is 1.99 Å) from the intercalation site (Fig. 6c) takes place. In the case of C_60_ + HL1, a similar effect is observed for C_60_ molecule (Rmsd value for HMF compound is 2.69 Å) (Fig. 6d).

HL2 + C_60_ or C_60_ + HL2*:* in these cases, there is a significant shift of the C_60_ fullerene with respect to the HL2 compound at their combined interaction with DNA in small and large grooves (Fig. 7a, b). So, Rmsd value for C_60_ fullerene in a small groove is 7.16 Å (for HL2—1.69 Å), and in a large groove—8.56 Å (for HL2—3.85 Å). However, a complete break of stacking interaction between C_60_ fullerene and HL2 does not happen.

At intercalation of the studied structures into a small groove of DNA, the C_60_ fullerene forms the strong stacking interactions with HL2 and nitrogenous bases of DNA double helix (Fig. 7c, d). So, after molecular docking, its shift is negligible: in the case of HL2 + C_60_, the Rmsd value for C_60_ fullerene is 3.99 Å (for HL2—1.36 Å), and in the case of C_60_ + HL2—2.7 Å (for HL2—3.29 Å). In addition, at such intercalation, there is no displacement of chemical compound from the original nucleotide environment.

As shown in Table 1, the energy parameters of C_60_ + HL1 or C_60_ + HL2 intercalation into DNA molecule are lower for C_60_ + HL1 structure than for HL1 + C_60_ or HL2 + C_60_ structures. The Bump value for C_60_ + HL1 structure is 17.1 kJ/mol and Int—4.2 kJ/mol, while for HL1 + C_60_ structure, these parameters are higher—19.6 and 7.2 kJ/mol, respectively. These data allow to suggest that the studied chemical compounds form more stable structures with C_60_ fullerene when C_60_ molecule is initially bound to DNA.

Taking into account the structural features of the studied compounds and C_60_ fullerene, one can suggest the formation of cation-π bonds between HL1 and C_60_ fullerene due to CCl_3_ group (Fig. 6a) and stacking interactions between HL2 and C_60_ fullerene mediated by the presence of benzene ring (Fig. 7a).

Thus, using the computer modeling, it was found that HL2 and HL1 compounds in combination with C_60_ fullerene are potentially capable of binding to DNA molecule.

## Conclusions

We have estimated effect of two carbacylamidophosphate derivatives with different substituents on the viability of leukemic cells of three lines. Dimorfolido-*N*-trichloroacetylphosphorylamide (HL1) and dimorfolido-*N*-benzoylphosphorylamide (HL2) at 1 mM concentration in the cellular medium caused the decrease of cell viability, which value was dependent on the cell type and duration of incubation. HL1 and HL2 caused the similar gradual decrease of Jurkat cells viability. HL1 had earlier and more profound toxic effect as compared to HL2 both on Molt-16 and CCRF-CEM cells. Toxic effect of HL2 was detected only at 72 h of Jurkat and Molt-16 cells incubation.

It was shown that C_60_ fullerene facilitated the toxic effect of HL2 on Jurkat and CCRF-CEM cells at 48 and 72 h.

By use of computer simulation, the interactions of DNA molecule with HL1 or HL2 separately and in combination with C_60_ fullerene were studied. The chemical compounds formed the stable complexes with DNA: HL1 when binding with a great groove and at intercalation into a small groove, while HL2 only when bound with a small groove. At the combined action of HL1 or HL2 with C_60_ fullerene, the C_60_ + HL2 structure formed the stable complex with DNA when bound with a small groove and at intercalation into it, while the C_60_ + HL1 structure formed the stable complex with DNA only in one case—when binding with a large groove. In this case, the strong stacking or cation-π interactions can be formed between these chemicals and C_60_ fullerene. Thus, the chemical compounds form the stable complexes with DNA both individually and in combination with C_60_ fullerene, but the differences in the types of bonds and ways of binding could be the cause of their different cytotoxic effects.

## Abbreviations

DMSO: Dimethyl sulfoxide
DNA: Deoxyribonucleic acid
FBS: Fetal bovine serum
HL1: Dimorfolido-*N*-trichloroacetylphosphorylamide
HL2: Dimorfolido-*N*-benzoylphosphorylamide
IR: Infrared spectroscopy
MTT: 3-(4,5-Dimethyl-2-thiazolyl)-2,5-diphenyl-2-H-tetrazolium bromide
NMR: Nuclear magnetic resonance
RPMI - 1640 medium: Roswell Park Memorial Institute medium
